# Impaired Sense of Smell in a *Drosophila* Parkinson’s Model

**DOI:** 10.1371/journal.pone.0073156

**Published:** 2013-08-29

**Authors:** Simone Poddighe, Krishna Moorthi Bhat, Maria Dolores Setzu, Paolo Solla, Anna Maria Angioy, Roberto Marotta, Roberta Ruffilli, Francesco Marrosu, Anna Liscia

**Affiliations:** 1 Department of Biomedical Sciences, University of Cagliari, Cagliari, Italy; 2 Department of Neuroscience and Cell Biology, University of Texas Medical Branch, Galveston, Texas, United States of America; 3 Department of Public Health, Clinical and Molecular Medicine, University of Cagliari, Cagliari, Italy; 4 Department of Experimental Biology, University of Cagliari, Cagliari, Italy; 5 Nanobiotech Facility, Italian Institute of Technology, Genova, Italy; 6 Department of Life and Environmental Sciences, University of Cagliari, Cagliari, Italy; University of Sheffield - MRC Centre for Developmental and Biomedical Genetics, United Kingdom

## Abstract

Parkinson’s disease (PD) is one of the most common neurodegenerative disease characterized by the clinical triad: tremor, akinesia and rigidity. Several studies have suggested that PD patients show disturbances in olfaction at the earliest onset of the disease. The fruit fly *Drosophila melanogaster* is becoming a powerful model organism to study neurodegenerative diseases. We sought to use this system to explore olfactory dysfunction, if any, in *PINK1* mutants, which is a model for PD. *PINK1* mutants display many important diagnostic symptoms of the disease such as akinetic motor behavior. In the present study, we describe for the first time, to the best of our knowledge, neurophysiological and neuroanatomical results concerning the olfactory function in *PINK1* mutant flies. Electroantennograms were recorded in response to synthetic and natural volatiles (essential oils) from groups of *PINK1* mutant adults at three different time points in their life cycle: one from 3–5 day-old flies, from 15–20 and from 27–30 days. The results obtained were compared with the same age-groups of wild type flies. We found that mutant adults showed a decrease in the olfactory response to 1-hexanol, α-pinene and essential oil volatiles. This olfactory response in mutant adults decreased even more as the flies aged. Immunohistological analysis of the antennal lobes in these mutants revealed structural abnormalities, especially in the expression of Bruchpilot protein, a marker for synaptic active zones. The combination of electrophysiological and morphological results suggests that the altered synaptic organization may be due to a neurodegenerative process. Our results indicate that this model can be used as a tool for understanding PD pathogensis and pathophysiology. These results help to explore the potential of using olfaction as a means of monitoring PD progression and developing new treatments.

## Introduction

Parkinson’s disease (PD) represents one of the most common neurodegenerative disorders and is usually described by the overt clinical motor triad: tremor, bradykinesia and rigidity. However, previous studies have demonstrated that several non-motor symptoms may precede the onset of motor impairment [1] [2]. Of these symptoms, a decreased olfactory function in PD is a common finding, which likely occurs early in the disease process [3]. Indeed, although under-detected in clinical practice, deficits in olfactory function may precede the onset of motor symptoms by approximately 4 years [4] [5]. According to neuropathological studies in humans [6], it is now clear that the PD-related intraneuronal pathology evolves through at least six progressive steps, which include the medulla oblongata and olfactory bulb to the midbrain, diencephalic nuclei and neocortex. In this context, it must be emphasized that the substantia nigra, whose impairment plays a key role in the motor impairment, is involved only in a later step in PD, while typical PD-related alterations can initially be observed in the anterior olfactory nucleus.

Recently, several genetic mutations have been described as important etiologic factors in the degeneration of dopaminergic neurons in the substantia nigra. In particular, mutations in the PTEN-induced putative kinase 1 (*PINK1*) gene [7] in humans are known to cause hereditary early-onset PD. While previous studies using a small cohort of PD patients with *PINK1* mutations showed deficits in odor identification and discrimination, the pathophysiology of olfactory dysfunction remains largely obscure [8] [9] [10].

Over the years the fruit fly *Drosophila* has been considered particularly useful as a model system in studying neuronal dysfunction in general, and investigating the early stages and molecular aspects of neurodegenerative diseases in particular. Although models can only heuristically fulfil reductive reproductions of human diseases, many of the existing PD models in *Drosophila* exhibit key features of the disease and have provided insights into PD pathogenesis. Among them, *PINK1* mutants provide capital clues regarding the pathogenetic molecular basis of PD because juvenile sporadic forms of PD share certain common pathways with that of *PINK1*-linked PD. *PINK1* mutants exhibit indirect flight muscle and dopaminergic neuronal degeneration accompanied by locomotor defects. This gene encodes a putative serine/threonine kinase with a mitochondrial targeting sequence [7], while a recent study demonstrates that the kinase domain faces the cytosol, where its physiological substrates may reside [11].

Olfactory dysfunctions are a common feature in *PINK1* Parkinsonism and consist typically of defective odor identification and discrimination both in human [8] [9] [10] and in animal PD models [12]. The study of *in vivo* models of *PINK1* mutants may represents an effective approach in evaluating olfactory alterations. Moreover, *PINK1* is an autosomal recessive gene associated with early onset of PD [7] and may represent an effective paradigm for detecting the development of the disease features rather than other genetic late onset models showing reduced penetrance in humans, as in the case of *LRRK2* mutations [13].

As the *Drosophila PINK1* gene encodes a protein that contains the same domains as its human counterpart, we sought to study alterations in olfactory functions in *Drosophila PINK1* mutants. In particular, we undertook to correlate early stages of clinical features of PD in human patients to our fly model.

In this study, we describe neurophysiological and neuroanatomical results concerning the olfactory function in *PINK1^B9^* mutant flies. We combined electrophysiological and morphological techniques and demonstrate that these flies show olfactory deficits as well as defective antennal lobes. The results show that the *PINK1^B9^* mutant adults have a lowered olfactory responsiveness to stimuli from the majority of the odors tested and an impairment of the integrity of presynaptic active zones in the olfactory areas in the CNS.

## Materials and Methods

### Fly Strains, Genetics

For the experiments we used adult males of the following *Drosophila melanogaster* strains: wild type and *PINK1^B9^* (from Bloomington stock center). After emergence from pupae, males of both strains were separated. Flies were reared on a standard cornmeal–yeast–agar medium in controlled environmental conditions (24–25°C; 60% RH; L:D = 12∶12). Standard genetic procedures were used during the study.

### Lifespan Determination

Males of wild type (n = 115) and *PINK1^B9^* (n = 310) were maintained separately. Five flies per vial were kept for analysis and were transferred every day to new tubes with fresh medium. Their lifespan was measured by counting the number of dead animals and expressed as survival rate in %. Statistical differences of survival curves were analyzed using the Kaplan–Meier test.

### Electrophysiology

*In vivo* electroantennogram recordings (EAG) were performed as described previously [14] [15]. Briefly, live adult *Drosophila* in the age ranges of 3- to 5 (group I), 15- to 20 (group II) and 27- to 30 days (group III) were singly inserted in a truncated pipette with the antennae protruding from the narrow end of the tip. The preparation was fixed with dental wax on a microscope slide and positioned under the viewer of an Olympus BX51WI light microscope (Olympus, Tokyo, Japan). Glass capillaries with a silver wire were filled with a conductive 0.15 M NaCl solution. The recording glass electrode was gently positioned on the tip of the antennal funiculus while the reference electrode was pierced ipsilaterally through the compound eye. The EAG signal was amplified with an AC/CD probe and then acquired with an IDAC-4 interface board (Syntech, Hilversum NL). A charcoal purified and humidified airflow was constantly blown over the antennae (speed 0.5 m/s), via a glass tube, placed approximately 1 cm from the antenna. The tip of a Pasteur pipette containing an odor-loaded filter paper (5 mm×25 mm) was inserted into a small hole in the glass tube. Odor stimulation was administered by injecting a puff of purified air (0.5 s at 10 mL/s airflow) through the pipette using the stimulus delivery controller (Syntech). Odor stimuli were prepared in increasing concentrations (0.1, 1 and 10% in volume) diluted in hexane. One µL of a stimulating solution was loaded on the filter paper. Odor stimuli were randomly applied, allowing a 3-min interval between successive stimulations to avoid receptor adaptation. Each series started and ended with a control stimulation (pure air) followed by a blank (solvent).

Odor stimuli, 1-hexanol, isoamyl acetate [16] and ethyl 3-hydroxybutyrate [17] were chosen from among synthetic chemicals according to their well-known stimulant activity in *D. melanogaster* and between essential oils extracted from Mediterranean plants with established antioxidant and anti-inflammatory effects: Rosemary (RM) [18] [19], Lentisk (LT) [20] and Myrtle (MT) [21]. The essential oils as stimuli were tested to determine the effects of their properties on our model. Among the selected chemicals, α-pinene was also chosen because of its high concentration of essential oil blends [22] [23] [24]. Recordings were made in 15 specimens for each strain and age-range group. Mean values of EAG amplitude were calculated and then analyzed by comparing the results obtained in *PINK1^B9^* flies with the age-matched wild type (WT) control group. The significance of differences was tested with a two-way ANOVA (post hoc test Least Significance Difference LSD) with a threshold level of statistical significance set at *P*<0.05. EAG results are represented in histograms with means values ± SEM.

### Immunohistochemistry

We used a monoclonal antibody against Bruchpilot nc82 tolabel the antennal lobes (ALs), the olfactory structures in the brain homologous to the olfactory bulbs in mammals. The nc82 staining is routinely used to delineate the glomeruli in the fruit fly [25] [26] and diptera in general [27] because of its restricted labeling of synapses within the neuropil. We obtained the best results using a modified protocol by Seki et al. [26]. Whole heads of both WT and *PINK1^B9^* mutant (age-range group I) were fixed with 4% paraformaldehyde in phosphate-buffered saline with addition of 0.25% Triton X (PBST, pH 7.2) for 3 hours at room temperature. Brains were then dissected out, washed 3 times for 20 min in PBST and then placed in a 5% normal goat serum (NGS) blocking solution in PBST for 1 hour at room temperature. The three 20 min washes were repeated before incubating brains in a mouse anti-nc82 (Hybridoma, University of Iowa, Iowa, IA, USA) antibody (1∶30 in PBST-NGS). After washes, brains were incubated in the secondary antibody coupled with Alexa 633 (Molecular Probes, Carlsbad, CA, USA) diluted 1∶200 in PBST-NGS. Each incubation lasted 48 hours at 4°C. After removing the PBST, brains were mounted on a standard microscope slide in a Vectashield Hard set medium, using a spacer ring (Secure-Seal imaging spacers, Sigma Aldrich, St. Louis, MO, USA) to protect them from pressure by the coverslip. To exclude autoflorescence of the preparations, a blank was also prepared following the same staining procedure and timing but avoiding the primary antibody.

### Confocal Microscopy

Preparations were viewed using a Leica TCS SP5 confocal microscope equipped with a 40× APO PLAN oil immersion objective. Labeled structures with Alexafluor 633 were excited with a supercontinuum white light laser at 631 nm and fluorescence was detected at a range of 640–710 nm. Ten specimens for both *PINK1^B9^* and WT were scanned to obtain stacks of 25–33 confocal images. All fly brains were scanned using these defined confocal settings consisting of identical detector gain, amplifier gain, amplifier offset, pinhole diameter, excitation (laser power), scan mode and speed (line scan) and frame size.

### Image Processing

Image analysis was performed on a standard Windows XP platform using a free version of Image J and Imaris 7.0 (Bitplane AG, Zurich, Switzerland). The volume of individual AL was rendered and measured. Each stack of images was binarized and a standard threshold was established on the blank preparation. A “region of interest” (ROI) was demarcated by hand in each optical section to encircle the entire surface of each AL, from top to bottom, to enclose its entirety. The grey-scale value, given as an arbitrary unit (pixels/cm^2^), was measured in each ROI to evaluate staining intensity. Mean values of the results obtained in *PINK1^B9^* and WT were then compared and statistical significance was evaluated with a one-way ANOVA (*P*<0.05).

### Western Blot Analysis

Four samples of eight heads of both wild type and *PINK1^B9^* (age range group I) were homogenized in 20 µL of 5× Laemmli buffer and incubated for 5 min at 95°C before fractionation by sodium dodecyl sulfate–polyacrylamide gel electrophoresis on 8% minigels (Mini Protean II; Bio-Rad, Hercules, CA). The separated proteins were transferred to a polyvinylidene difluoride membrane (Bio-Rad), 100 V for 1 h (transfer buffer: Trizma-base 190 mM, Glycine 25 mM, Methanol 20%, v/v) and subjected to immunoblot analysis with mouse monoclonal antibodies to nc82 (1∶100 dilution). The membrane was incubated with primary antibodies overnight at 4°C, and immune complexes were detected with horseradish peroxidase–conjugated secondary antibodies and chemiluminescence reagents (ECL, Amersham Biosciences). The amount of bruchpilot protein was quantified by analysis of the corresponding bands on the autoradiogram with a densitometer (Geliance, Perkin Elmer). Data were normalized by dividing the optical density of the bands corresponding to Bruchpilot protein by that of the band for α-Tubulin (loading control), which was revealed by reprobing the membrane with rabbit monoclonal antibodies to Tubulin. Mean values of the results obtained in *PINK1^B9^* and WT were then compared and statistical significance was evaluated with a one-way ANOVA (*P*<0.05).

### Transmission Electron Microscopy

WT and *PINK1^B9^ Drosophila* (age range group I) were anesthetized with carbon dioxide and carefully decapitated. Brains were rapidly dissected out and fixed in a mixture of 2% glutaraldehyde and 2% paraformaldehyde in 0.1 M cacodylate buffer. Brains were then washed several times in the same buffer, post-fixed in 1% osmium tetroxide in H_2_O for 2 h and stained overnight at 4°C in an aqueous 0.5% uranyl acetate solution. Samples were finally washed several times in distilled water, dehydrated in a graded ethanol series and then embedded in SPURR resin.

To underline the ALs, brains were sliced into semi-thin coronal sections (∼0.5 µm) with a Leica EM UC6 ultramicrotome and then stained with toluidine blue and observed with a Leica DM2700 P light microscope. Sections of about 70 nm corresponding to the portions of the ALs were cut with a diamond knife on a Leica EM UC6 ultramicrotome. Images were obtained with a Jeol JEM 1011 electron microscope working at an acceleration voltage of 100 kV and acquired with an 11 Mp charge-coupled device camera (Gatan Orius SC100). Preparations were observed and the percentage of presynaptic boutons without mitochondria in both WT and *PINK1^B9^* was assessed. A total of 104 ALs micrographs were randomly sampled and observed for more than 100 synapses analyzed. Data were analyzed with Origin 8 software (OriginLab Co., Northampton, MA, USA). Mean values of the results obtained in WT and in mutants were compared and statistically evaluated with an “*t*” test. The threshold level of statistical significance was set at p<0.01.

### Behavior

Free-walking bioassays were performed following the experimental procedures used by Dekker et al [28]. Briefly, males of WT and *PINK1^B9^* (age range group II) were given the opportunity to choose between vials with water with or without odor. Two 4 mL glass vials were placed symmetrically and equally spaced in a large petridish (the arena) and then fitted with truncated pipette tips. The vials were filled with 300 µL of water with 0.25% Triton X with or without the odorant. The odor chosen to trap the flies was isoamyl acetate at the dilution of 0.1%. The dehydration of flies was prevented by placing a cotton ball with 3 mL of water in the arenas. Flies were starved for 8 hr prior to starting the experiments. The assays were performed in controlled environmental conditions and lasted 18 hr. The attraction index (AI) was calculated as follows: (T–C)/(T+C+NR–D), in which T is the number of flies in the treatment, C the number in the control, NR the number remaining in the arena and D the number of dead flies in the arena (n = 9 of bioassay for each strain of flies; n = 20 of flies per arena). Data obtained were statistically evaluated with a “*t”* test.

## Results

### *PINK1^B9^* Mutants Display Shortened Longevity

**Figure 1:**
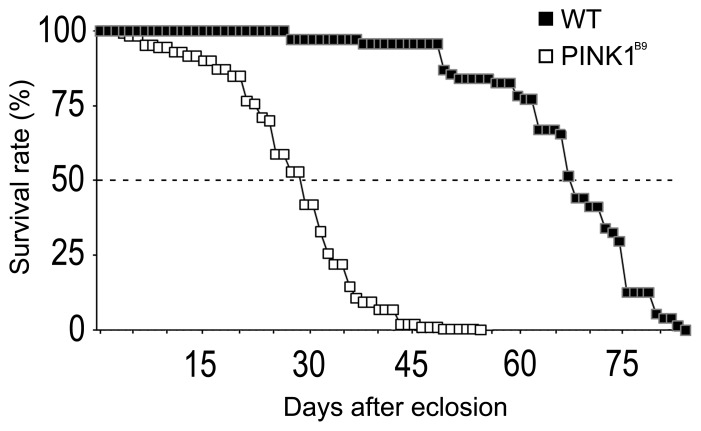
Lifespan in wild type and *PINK^B9^* mutant adults. The graph shows the survival rate observed in wild type and *PINK1^B9^* mutants. *PINK1^B9^* had a reduced lifespan compared to WT.*PINK1^B9^* flies started to die dramatically at the 15^th^ days after eclosion with 50% of flies being dead after 30 days (P<0.0001).

To determine the longevity of *PINK1* mutant flies, individuals were examined for their life span and compared to WT flies. These results are shown in Fig. 1. As shown in the figure, *PINK1^B9^* mutants displayed a shorter life span compared to WT (P<0.0001). *PINK1^B9^* flies started to die dramatically about 15 days after eclosion. The maximum number of days a mutant fly lived was about 53 days. These data show a reduced lifespan for *PINK1^B9^* mutants as was previously reported by Imai et al. [29].

### *PINK1^B9^* Mutants Show Odor-specific Reduced Electrophysiological Response

**Figure 2:**
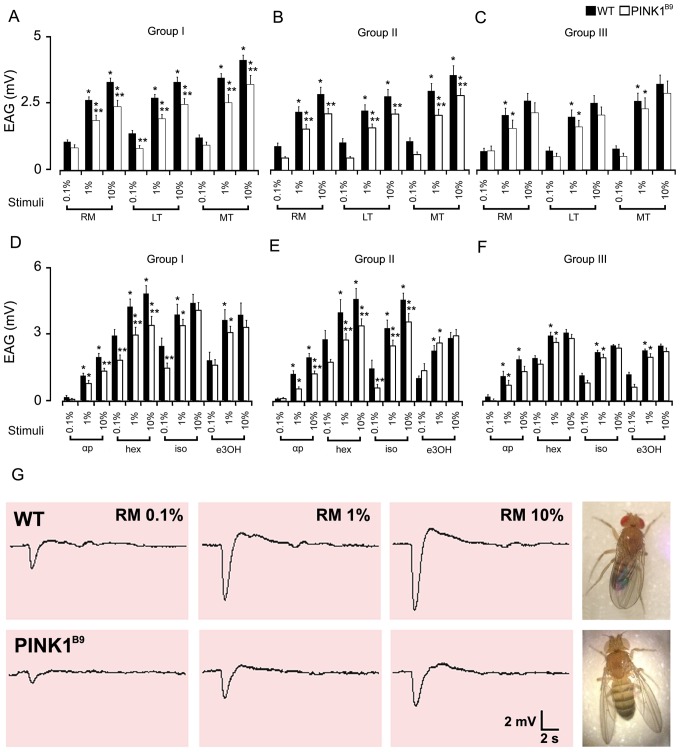
Electroantennogram responses in wild type and *PINK1^B9^* mutants. Dose-response relationships for olfactory stimulations in WT and *PINK1^B9^* adult flies and their differences in signal amplitude. Histograms in A and D show the EAG results in flies from Group I (age range from 3 to 6 days), from Group II (age range from 15 to 20 days) in B and E and from Group III (age range from 27 to 30) in C and F. Values shown are mean ± S.E.M. of the EAG amplitude. Stimuli are dilutions of essential oils (rosemary, RM; lentisk, LT; myrtle, MT) and synthetic compounds (α-pinene, αp; hexanol, hex; isoamyl acetate, iso; ethyl 3-hydroxybutyrate, e3OH) administered in a 3-step dose from 0.1 to 10% in hexane. EAGs obtained in WT and *PINK1^B9^* displayed strong similarities in the dose-response to stimuli; statistically significant differences were observed between WT and the mutant strains (^*^ significantly different from its previous concentration; ^**^ significantly different from its matching stimulus; *P*<0.05). In G, representative EAG tracings recorded in WT (upper) and *PINK1^B9^* (lower) in response to olfactory stimulation with rosemary oil (RM) administered in increasing concentrations. The sample tracings show that the amplitude of the depolarization in the baseline is clearly higher in WT than in *PINK1^B9^*.

We next sought to determine if *PINK1^B9^* mutant adults exhibited reduced electrophysiological response in an odor-specific manner. We found that the olfactory stimulations consistently elicited a typical waveform EAG response with rapid depolarization followed by a slow recovery phase in both WT and in *PINK1^B9^* mutant adults. The EAG values recorded in WT from e age group I were on the average higher than those obtained in mutant flies from the matching age group (P<0.05). Lower EAG values were measured when mutants were stimulated both with the oils and the synthetics. In detail the olfactory responses recorded from 15 specimens for both WT and *PINK1^B9^* individuals in age group I exhibited a dose response in both strains for all the stimuli administered. Thus, all animals responded with a higher EAG amplitude when stimulated with increased concentrations of odors. The results are summarized in Figs. 2A and 2D. The EAG signal amplitudes evoked by stimuli were significantly lower in *PINK1^B9^* than in WT for the majority of odors administered at the mid- and highest concentrations (*P*<0.05), with the exception of LT, which also displayed a significantly lower amplitude at the lowest concentration. Among the synthetic odors tested, the EAGs evoked by isoamyl acetate and ethyl 3-hydroxybutyrate in *PINK1^B9^* did not result significantly lower than those detected in WT, even displaying an undersized amplitude. The only exception was for the stimulation with isoamyl acetate at the lowest concentration, where EAGs in *PINK1^B9^* flies had a significantly lower amplitude than those in WT (*P*<0.05). Sample tracings of EAG recorded in WT and in *PINK1^B9^* mutant adults in response to stimulation with RM essential oil at increasing concentrations are shown in Fig. 2G, where the signal amplitude in WT is clearly higher compared to mutants.

To see if impairment of responses to stimuli progressed with age in *PINK1^B9^* mutants, the experiments were repeated using groups of middle-aged (group II) and older flies (group III), since *PINK1^B9^* mutants began to die at around the 15^th^ day with 50% of the flies being dead after 30 days (Fig. 1). These results are shown in Fig. 2B, E for the group II and in 2C, F for the group III. The results indicated that both strain of flies suffered from a general decrease in the average response correlated with aging (*P*<0.05). In fact, the average EAG amplitudes measured in flies from group II and III were lower than the values recorded in flies from group I. Despite the progressive decrease in olfactory response, the EAG amplitudes in WT from groups I, II and III were constantly higher than those in mutants of the age-matched groups (*P*<0.05). Interestingly, the *PINK1^B9^* flies, with respect to WT, displayed a different development of their decreasing trend in olfactory sensitivity with aging. Mutants in fact, which in any case displayed a lowered basal EAG, had the general significant decrease of sensitivity when they reached middle age (group II). The average EAG values measured in older *PINK1^B9^* flies from group III did not change substantially from those recorded in mutant flies from group II, thus sharing similarities and lacking statistical significance when compared.

In detail, the EAGs recorded in both strains of flies from group II (Fig. 2B, E) shared a similar trend for dose response dependence as observed in flies from group I. The difference between the two strains in response to odor stimuli was still present and almost unaltered. The only exception was with isoamyl acetate; significant differences were observed between the mutant and the wild type adults at all concentrations tested.

The general decreasing trend in sensitivity described above for WT of group III is clearly appreciable as shown in Figs. 2C and 2F, when the stimulation with the highest concentration of odors was never enough to elicit a significant higher amplitude in the EAGs, except for α-pinene which continued to evoke a plain dose response. When WT aged the differences in *PINK1^B9^* of the matched-age group tended to decrease, although WTs still maintained a higher average value (*P<*0.05). In *PINK1^B9^* even the dose response relationship appear to be altered as in WT, since neither of the stimuli elicited a significant EAG amplitude when administered at the highest concentration. The average values were not significantly different from those recorded in mutants of group II.

### Antennal Lobes in *PINK1* Mutant Adults Show Reduced Expression of Bruchpilot Protein

**Figure 3:**
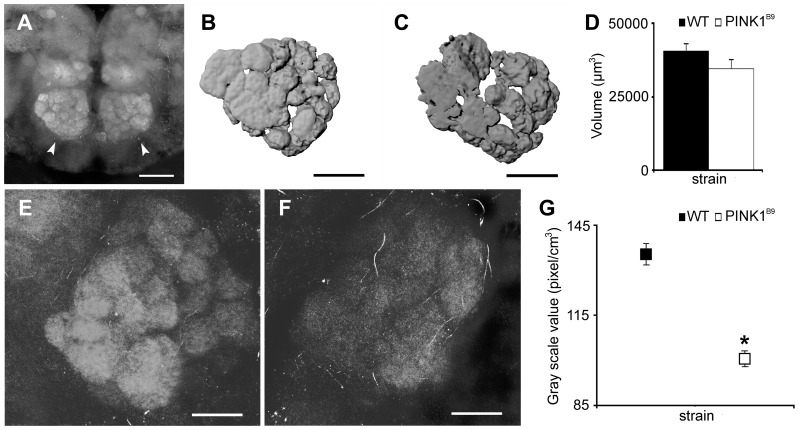
The antennal lobes reveal abnormalities in *PINK1^B9^* mutants. ALs were stained for the expression of Bruchpilot protein. Panel A: Confocal micrographs of a frontal view of a couple of ALs (indicated by the arrow heads), with their well-defined glomeruli in WT and the mutant (Scale bar = 50 µm). **B,C:** The volume rendering for the monoclonal nc82 antibody-stained AL as seen from a frontal view in a WT (B) and in a *PINK1^B9^* mutant (C) (scale bars = 30 µm). Ten specimens in the age range of 3–10 days for each strain were analyzed; the volumes measured were averaged and the statistical differences evaluated. Mean values ± S.E. are reported in C: no significant difference was detected (*P*>0.05). E,F: Higher magnification photomicrographs of a single AL in WT and *PINK1^B9^* mutant respectively (scale bars = 20 µm). The latter reproducibly displays a less intensive staining and the glomeruli are not as clearly defined as in the WT. The gray scale value, taken as the index of intensity, was measured on ALs from binarized stacks of images (n = 10 for each strain). Mean values ± S.E. for both WT and *PINK1^B9^* mutants are shown in G. Statistical evaluation of the data shows that the staining intensity in *PINK1^B9^* is significantly lower compared to WT; (*significantly different from its matching value; *P*<0.05).

To see if the defects observed in the electroantennograms can be correlated with any defects in the antennal lobes (ALs) in the brain, we performed immunostaining of brains from *PINK1^B9^* mutants and compared the pattern to wild type brains. We used the monoclonal antibody nc82 for this purpose. The monoclonal nc82 antibody was raised against the Bruchpilot protein, which is a marker for active zones in synapsis. The labelling of the synapses with nc82 showed the pair of ALs located frontally in the brain, as well as their sets of glomeruli (Fig. 3A). The analysis performed on the stacks of images acquired with confocal microscopy allowed us to obtain volume renderings of the ALs. Ten specimens for both WT and *PINK1^B9^* were analyzed and their corresponding average size in µm^3^ was measured. These results are shown in Fig. 3B for WT and Fig. 3C for *PINK1^B9^* mutants. The results obtained revealed no particular divergence in the shape or in volume quantification of ALs. The volume values measured were 40636±2556 for WT and 34619±3130 µm^3^ for *PINK1^B9^*and no statistical significance was found between the two strains (Fig. 3D).

Examples of stained ALs in WT and *PINK1^B9^* adults are shown in Figs. 3E and 3F respectively. In *PINK1^B9^* mutants, as is clearly seen in the image, the staining was less intensive and the ALs were much less bright and less defined compared to WT. This result suggests a reduced expression of the Bruchpilot protein. The graph in Fig. 3G summarizes the results for the quantification of nc82 staining intensity in ALs. The gray scale values measured for each AL in the demarcated ROI were averaged for each strain and then compared. These reveal a significantly lower intensity value for *PINK1^B9^* (*P*<0.05) compared to WT.

**Figure 4:**
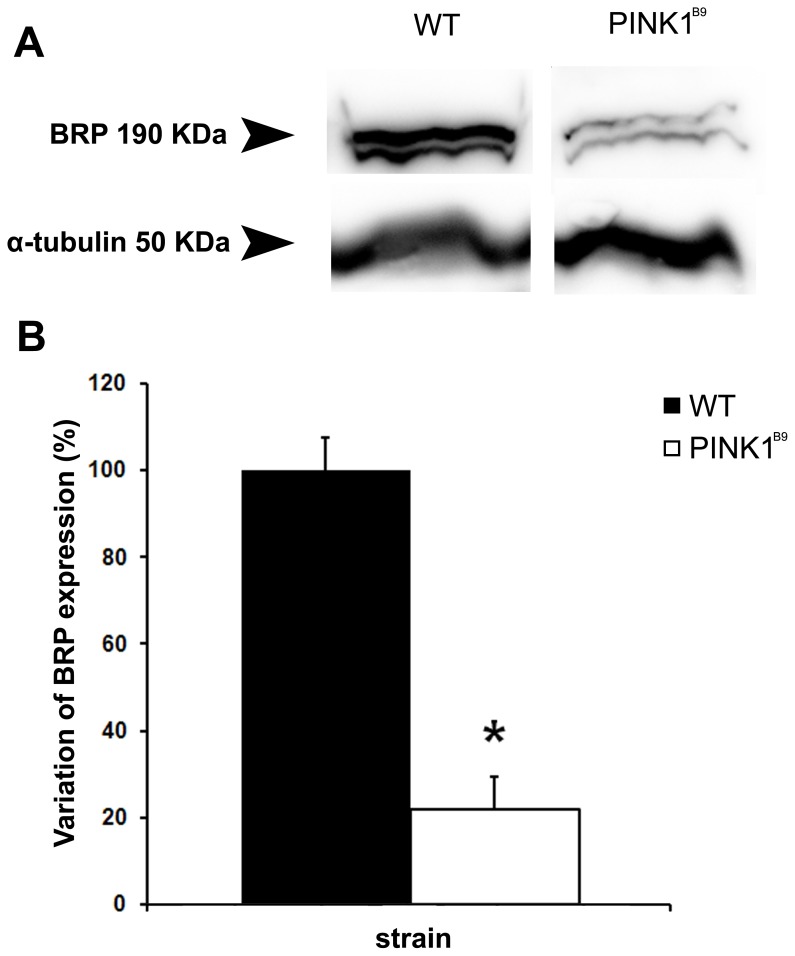
Western blot analysis of *PINK1^B9^* heads shows a reduced expression of the bruchpilot protein. **A:** Western blot analysis of adult head homogenate from WT and *PINK1^B9^* flies showing the nc82-labeled Bruchpilot protein band (BRP, top) and the loading control Tubulin (bottom). The amount of Bruchpilot protein was quantified by analyzing the intensity of the bands on the autoradiogram with a densitometer. Data were normalized by dividing optical density of the bands corresponding to Bruchpilot protein by that of the band for α-Tubulin. **B:** Statistical evaluation of the densitometric data shows that the expression of Bruchpilot protein in *PINK1^B9^* is significantly lower compared to WT (*significantly lower than its matching value; *P*<0.05).

To see if the expression of the Bruchpilot protein was affected in *PINK1^B9^* brains, we performed Western blot analysis of WT and *PINK1^B9^* head homogenate using nc82 (Fig. 4A). The comparison of densitometric data obtained by analysing the corresponding bands showed that there is a significant reduction in the amount of the Bruchpilot protein in *PINK1^B9^*. The average overall expression of Bruchpilot in *PINK1^B9^* was about 21.9% of the wild type, significantly lower than that of WT (*P*<0.002). These results are shown in the histogram in Fig. 4B. It is known that proper expression of this protein is critical for the structural integrity of synaptic active zones and for normal-evoked neurotransmitter release in *Drosophila* [30]. This reduced expression therefore indicates that the ALs in *PINK1^B9^* mutants have likely suffered damage to the structural integrity of synaptic active zones and perhaps underlie their defective odor perception (see Discussion).

### In the Antennal Lobes of *PINK1^B9^* the Mitochondria within the Presynaptic Boutons were Altered

**Figure 5:**
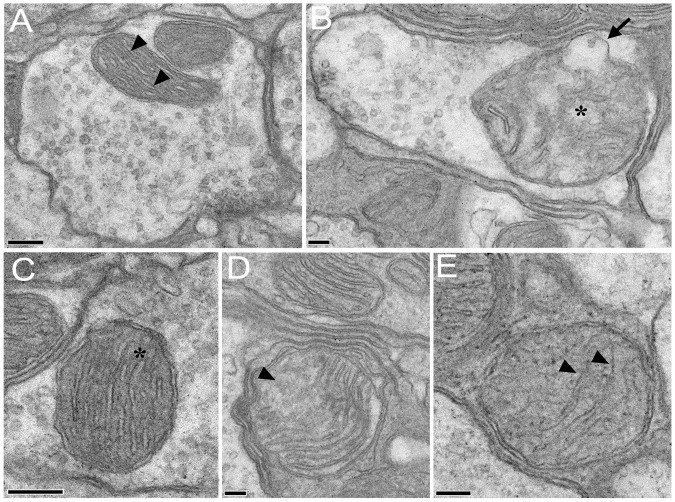
Mitochondria within the presynaptic boutons in the antennal lobes present structural alterations in *PINK1^B9^*mutants. Transmission electron microscopy (TEM) images of mitochondria within the olfactory bulbs of WT and *PINK1^B9^ Drosophila*. A: Two mitochondria in a presynaptic bouton of a WT (scale bar = 200 µm). The regular array of mitochondrial cristae (arrowheads) is surrounded by an electron-dense matrix. B: A mitochondrion in a presynaptic bouton of a *PINK1^B9^* mutant (scale bar = 100 µm). The mitochondrion presents a swelling on its external membrane (arrow) and the mitochondrial cristae are highly degenerated (asterisk). C: a conventional mitochondrion in a WT (scale bar = 200 µm). The regular electron-dense matrix surrounds the mitochondrial cristae (asterisk). D: The abnormal mitochondrial morphology in a *PINK1^B9^* mutant. The mitochondrial cristae are fragmented (arrowhead). E: The abnormal mitochondrial morphology in a *PINK1^B9^* mutant (scale bar = 100 µm). Remnants of the mitochondrial cristae are visible within the mitochondrial matrix (scale bar = 100 µm).

The mitochondria within the presynaptic boutons of *PINK1^B9^* ALs appeared clearly degenerated in comparison with those in WT (Fig. 5), being swollen and often presenting wide swellings on their outer membrane (Fig. 5B). The mitochondrial cristae, clearly fragmented and in some cases completely deteriorated, appeared surrounded by a highly altered, inhomogeneous electron transparent mitochondrial matrix (compare Fig. 5A, B with C, D). Furthermore we observed a significantly higher number of presynaptic boutons without mitochondria in *PINK1^B9^* than in WT specimens (Fig. 5E). These results suggest that the loss of function of the *PINK1* gene induced degeneration of mitochondria also within the presynaptic boutons in the ALs.

### Behaviour

To better understand if, and in what way, olfactory behaviour may be affected in *PINK1^B9^* flies, we performed a series of free-walking bioassays. Our observations (data not shown) revealed an AI score of 0.4±0.05 and 0.5±0.3 (mean ± SEM) in WT and *PINK1^B9^* respectively. The result obtained was not statistically significant (p = 0.6).

## Discussion

The aim of this study was to determine if there is any olfactory impairment in the *PINK1^B9^* mutant both in peripheral olfactory organs and in the organization of olfactory areas in the brain. To the best of our knowledge, this is the first study to examine possible olfactory deficits using *Drosophila* models. The data obtained are of particular interest given that it is in a scarcely investigated area of preclinical PD. The olfactory approach to this issue appears to be especially promising in view of its potential role in early diagnosis of premotor PD in human patients. Our results add to the debate on the role of olfactory measurements in predicting PD [31]. This work supports the idea that olfactory measurements may be a valid tool in the diagnosis of premotor PD. Our work also indicates that the molecular basis for such defects may lie within the ALs with structural defects, either developmental or post-developmental, affecting synaptic connectivity and or transmission.

An EAG represents the summed activity of the sensory receptor neurons in the antennae [32], which in flies is the equivalent of a nose. The EAG recordings indicate a reduction in the response to various odor stimuli. A significant decrease in sensitivity was detected in *PINK1^B9^* after stimulation with α-pinene and 1-hexanol, and all the essential oils tested, thus indicating a general loss of sensitivity to odors. Furthermore, a significant decrease in sensitivity was detected when stimulating mutant adults with isoamyl acetate, thus indicating a progressive loss of sensitivity to odors with age. These results confirm the importance to establishing the right test stimuli for the olfactory measurement test. In fact, the data available in the literature often disagree on what should be the right test stimuli. In any case, n-butanol, is considered one of the best stimuli for screening human patients [33].

We did expect to detect a much more pronounced worsening in the olfactory response with age in *PINK1^B9^* compared to the wild type, especially given that the colony was halving between 15–30 days from eclosion. However, while there was a small reproducible difference with older animals, the difference was statistically not significant. Therefore, we conclude that it is unlikely that *PINK1* mutants significantly suffer a greater loss of sensitivity with age compared to WT.

On the other hand, data obtained through immunohistochemistry together with the Western blot analysis are complementary to the electrophysiological findings. Indeed, *PINK1^B9^* mutant flies with reduced sensitivity to olfactory stimuli showed less defined nc82 staining, as revealed by quantification of staining intensity in their ALs and quantification of Bruchpilot protein expression. This result therefore indicates an altered expression of the Bruchpilot protein. Our Western analysis data were also consistent with *PINK1* affecting Bruchpilot protein expression. It is known that proper expression of Bruchpilot is critical for maintaining structural integrity of synaptic active zones and for normal-evoked neurotransmitter release in *Drosophila* [32]. Finally, those findings were completed by the observation of the degenerative alterations of the presynaptic boutons in *PINK1^B9^* as revealed by the transmission electron microscopic analysis. The active presynaptic zones are actually the termination of the cholinergic [34] olfactory sensory neurons (OSNs) that project to the ALs. Our results show that in *PINK1^B9^* mutants these zones are significantly damaged. The degeneration of OSNs likely also leads to a loss of functionality, e.g. an impaired synthesis of receptor proteins, which was revealed by the lowered average EAGs amplitudes recorded in *PINK1^B9^*. Mitochondrial degeneration may also lead to the underexpression of Bruchpilot.

Studies on the causes of olfactory impairment in humans are often controversial since it is difficult to study *in vivo* and only by imaging techniques or post-mortem analysis. In a study by Rombeaux et al [35] the reduction of olfactory bulbs (OBs) volume is considered a feature of peripheral olfactory damage. In fact, they found a correlation between peripheral olfactory loss and reduction in OBs volume, the latter declining in parallel with the olfactory function. In the case of PD patients, the progressive decline in the sense of smell does not appear to affect the volume of OBs [36]. On However, a more recent study observed a significant decrease in OBs volume and an impairment of the sense of smell in their panel of PD patients [37] [38]. Our results on volumetric measurements of ALs indicate that *PINK1* mutants had a lower volume compared to WT, although the difference was not statistically significant. However, we think that the alteration in the olfactory synaptic structures may be responsible for modifying the olfactory perception and behaviour in the *PINK1* mutant. Concerning the behavior, our data indicated that the attraction index (AI) scored by WTs and mutants were not different. However, the AI scored by the mutants appeared not homogeneous with a high inner variability. Despite of an apparently higher AI scored by mutants, on the average more WT (∼39% vs ∼33%) were trapped in stimulus. These data may be explained by taking into account that normal sensory perception, and thus odor evoked behaviour, is based not only on an input from OSNs but mostly on a balance between local excitatory and inhibitory interneurons within the ALs [39]. It was demonstrated, by means of genetic tools, that the suppression or reduction of a subset of synapses, mostly inhibitory, converted the response to odors as repulsive, while the suppression of another subset of mostly excitatory synapses shifted the perceptions of odorants to attraction. In other words, those previous reports suggest that *PINK1^B9^* mutants may suffer from a certain impairment in the normal balance of local interneurons within the ALs.

## Conclusions

Our functional approach to merging sensory electrophysiology with morphology of olfactory areas in the brain is unique in that a correlation between the two will greatly assist in our understanding of odor deficits in PD that may be used to help in diagnosing and treating PD patients. This approach can perhaps be used to evaluate and understand other neurodegenerative diseases such as Alzheimers and Huntington’s. At the same time, our data obtained with the EAG and the morphological analysis show the impairment of the olfactory function detectable both peripherally and in the CNS, and strongly suggest that the altered synaptic organization represents a neurodegenerative process correlated with mitochondrial dysfunction caused by the mutation of the gene *PINK1* [40]. Nonetheless, our results confirm that the *Drosophila* PD model *PINK1^B9^* represents a powerful tool not only in examining the pathogenesis and pathophysiology of PD, but also an important first step in discriminating new therapeutic approaches using olfaction as a criterion and which could be used to effectively monitor progression of PD in humans, inasmuch as the olfactory disturbance is often nothing but a warning of a general inner disease.

## References

[1] Koller WC, Busenbark K, Gray C, Hassanein RS, Dubinsky R (1992) Classification of essential tremor. Clin Neuropharmacol 15: 81–87. 1591741 10.1097/00002826-199204000-00001

[2] Wolters EC, Francot C, Bergmans P, Winogrodzka A, Booij J, et al (2000) Preclinical (premotor) Parkinson’s disease. J Neurol 247: 103–109. 10991655

[3] Doty RL, Deems DA, Stellar S (1988) Olfactory dysfunction in parkinsonism: a general deficit unrelated to neurologic signs, disease stage, or disease duration. Neurology 38: 1237–1244 doi:10.1212/WNL.38.8.1237 3399075 10.1212/wnl.38.8.1237

[4] Berendse HW, Ponsen MM (2006) Detection of preclinical Parkinson’s disease along the olfactory trac(t). J Neural Transm 70: 321–325. 10.1007/978-3-211-45295-0_48 17017547

[5] Haehner A, Hummel T, Reichmann H (2011) Olfactory Loss in Parkinson’s Disease. Parkinsons Dis 2011: 1–6 doi:10.4061/2011/450939 10.4061/2011/450939 PMC3109349 21687752

[6] Braak H, Del Tredici K, Rub U, De Vos RA, Jansen Steur EN, et al (2003) Staging of brain pathology related to sporadic Parkinson’s disease. Neurobiol Aging 24: 197–211. 12498954 10.1016/s0197-4580(02)00065-9

[7] Valente EM, Abou-Sleiman PM, Caputo V, Mugit MMK, Harvey K, et al (2004) Hereditary early-onset Parkinson’s disease caused by mutation in PINK1.Science 304. 1158: 1160 doi:10.1126/science.1096284 10.1126/science.1096284 15087508

[8] Ferraris A, Ialongo T, Passali GC, Pellecchia MT, Brusa L, et al (2009) Olfactory Dysfunction in Parkinsonism Caused by PINK1 Mutations. Mov Disord 24: 2350–2357. 19890973 10.1002/mds.22816

[9] Eggers C, Schmidt A, Hagenah J, Brüggemann N, Klein JC, et al (2010) Progression of subtle motor signs in PINK1 mutation carriers with mild dopaminergic deficit. Neurology 74: 1798–1805 doi:10.1212/WNL.0b013e3181e0f79c 20513816 10.1212/WNL.0b013e3181e0f79c

[10] Kertelge L, Brüggemann N, Schmidt A, Tadic V, Wisse C, et al (2010) Impaired sense of smell and color discrimination in monogenic and idiopathic Parkinson’s disease. Mov Disord 25: 2665–2669 doi:10.1002/mds.23272 20721915 10.1002/mds.23272

[11] Zhou C, Huang Y, Shao Y, May J, Prou D, et al (2008) The kinase domain of mitochondrial PINK1 faces the cytoplasm. Proc Natl Acad Sci U S A. 19 105: 12022–12027. 10.1073/pnas.0802814105 PMC2575334 18687899

[12] Glasl L, Kloos K, Giesert F, Roethig A, Di Benedetto B, et al (2012) Pink1-deficiency in mice impairs gait, olfaction and serotonergic innervation of the olfactory bulb. Experimental neurology 235: 214–227 doi:10.1016/j.expneurol.2012.01.002 22265660 10.1016/j.expneurol.2012.01.002

[13] Latourelle JC, Sun M, Lew MF, Suchowersky O, Klein C, Golbe LI, et al (2008) The Gly2019Ser mutation in LRRK2 is not fully penetrant in familial Parkinson’s disease: the GenePD study. BMC medicine 5: 6–32 doi:10.1186/1741-7015-6-32 10.1186/1741-7015-6-32 PMC2596771 18986508

[14] Ibba I, Angioy AM, Hansson BS, Dekker T (2010) Macroglomeruli for fruit odors change blend preference in Drosophila. Naturwissenschaften 97: 1059–1066 doi:–––10.1007/s00114–010–0727–2 20972770 10.1007/s00114-010-0727-2

[15] Poddighe S, Dekker T, Scala A, Angioy AM (2010) Olfaction in the female sheep botfly. Naturwissenschaften 97: 827–835 doi:–––10.1007/s00114–010–0700–0 20665207 10.1007/s00114-010-0700-0

[16] Keller A, LB Vosshall (2007) Influence of odorant receptor repertoire on odor perception in humans and fruit flies. PNAS 104: 5614–5619. 17372215 10.1073/pnas.0605321104 PMC1838502

[17] Hansson BS, Knaden M, Sachse S, Stensmyr MC, Wicher D (2009) Towards plant-odor-related olfactory neuroethology in Drosophila. Chemoecology 20: 51–61 doi:–––10.1007/s00049–009–0033–7 20461131 10.1007/s00049-009-0033-7 PMC2864897

[18] Celiktas OY, Bedir E, Sukan FV (2007) In vitro antioxidant activities of Rosmarinus officinalis extracts treated with supercritical carbon dioxide. Food Chemistry 101: 1474–1481.

[19] Schwarz K, Ternes W (1992) Antioxidative constituents of Rosmarinus officinalis and Salvia officinalisI. Determination of phenolic diterpenes with antioxidative activity amongst tocochromanols using HPLC. Z Lebensm Unters Forsch 195: 95–98. 1529647 10.1007/BF01201765

[20] Ljubuncic P, Song H, Cogan U, Azaizeh H, Bomzon A (2005) The effects of aqueous extracts prepared from the leaves of Pistacia lentiscus in experimental liver disease. J Ethnopharmacol 100: 198–204. 16054533 10.1016/j.jep.2005.03.006

[21] Tuberoso CIG, Rosa A, Bifulco E, Melis MP, Atzeri A, et al (2010) Chemical composition and antioxidant activities of Myrtus communis L. berries extracts. Food Chem 123: 1242–1251 doi:10.1016/j.foodchem.2010.05.094

[22] Congiu R, Falconieri D, Marongiu B, Piras A, Porcedda S (2002) Extraction and isolation of Pistacia lentiscus L. essential oil by supercritical CO2. Flavour Fragr J 17: 239–244 doi:10.1002/ffj.1095

[23] Moghtader1 M, Salari H, Farahmand A (2011) Evaluation of the antifungal effects of rosemary oil and comparison with synthetic borneol and fungicide on the growth of Aspergillus flavus. J Ecol Nat Environ 3: 210–214.

[24] Zanetti S, Cannas S, Molicotti P, Bua A, Cubeddu M, et al (2010) Evaluation of the antimicrobial properties of the essential oil of Myrtus communis L. against clinical strains of Mycobacterium spp. Interdiscip Perspect Infect Dis 2010: 1–3 doi:10.1155/2010/931530 10.1155/2010/931530 PMC2914267 20706606

[25] Laissue PP, Reiter C, Hiesinger PR, Halter S, Fishback KF, et al (1999) Three-Dimensional Reconstruction of the Antennal Lobe in Drosophila melanogaster. J Comp Neurol 405: 543–552. 10098944

[26] Seki Y, Rybak J, Wicher D, Sachse S, Hansson BS (2010) Physiological and morphological characterization of local interneurons in the Drosophila antennal lobe. J Neurophysiol 104: 1007–1019 doi:10.1152/jn.00249.2010 20505124 10.1152/jn.00249.2010

[27] Ignell R, Dekker T, Ghaninia M, Hansson BS (2005) Neuronal architecture of the mosquito deutocerebrum. J Comp Neurol 493: 207–240 doi:10.1002/cne.20800 16255032 10.1002/cne.20800

[28] Dekker T, Ibba I, Siju KP, Stensmyr MC, Hansson BS (2006) Olfactory shifts parallel superspecialism for toxic fruit in Drosophila melanogaster sibling, D. sechellia. Curr Biol 16: 101–109 doi:10.1016/j.cub.2005.11.075 16401429 10.1016/j.cub.2005.11.075

[29] Imai Y, Kanao T, Sawada T, Kobayashi Y, Moriwaki Y, et al (2010) The Loss of PGAM5 Suppresses the Mitochondrial Degeneration Caused by Inactivation of PINK1 in Drosophila. PLoS Genet 6(12): e1001229 doi:10.1371/journal.pgen.1001229 21151955 10.1371/journal.pgen.1001229 PMC2996328

[30] Wagh DA, Rasse TM, Asan E, Hofbauer A, Schwenkert I et al.. (2006) Bruchpilot, a Protein with homology to ELKS/CAST is required for structural integrity and function of synaptic active zones in Drosophila. Neuron 49: 833–844. doi 10.1016/j.neuron.2006.02.008. 10.1016/j.neuron.2006.02.008 16543132

[31] Haehner A, Boesveldt S, Berendse HW, Mackay-Sim A, Fleischmann J, et al (2009) Prevalence of smell loss in Parkinson’s disease– a multicenter study. Parkinsonism Relat Disord 15: 490–494. 19138875 10.1016/j.parkreldis.2008.12.005

[32] Schneider D (1962) Electrophysiological investigation on the olfactory specificity of sexual attracting substances in different species of moths. J Insect Physiol 8: 15–30.

[33] Hummel T, Sekinger B, Wolf SR, Pauli E, Kobal G (1997) Sniffin’ sticks’: olfactory performance assessed by the combined testing of odor identification, odor discrimination and olfactory threshold. Chem Senses 22: 39–52. 9056084 10.1093/chemse/22.1.39

[34] Kazama H, Wilson RI (2008) Homeostatic matching and nonlinear amplification at identified central synapses. Neuron. 8: 401–413 doi:10.1016/j.neuron.2008.02.030 10.1016/j.neuron.2008.02.030 PMC2429849 18466750

[35] Rombaux P, Duprez T, Hummel T (2009) Olfactory bulb volume in the clinical assessment of olfactory dysfunction. Rhinology 47: 3–9. 19382487

[36] Mueller A, Abolmaali ND, Hakimi AR, Gloeckler T, Herting B, et al (2005) Olfactory bulb volumes in patients with idiopathic Parkinson’s disease – a pilot study. J Neural Transm 112: 1363–1370 doi:10.1007/s00702-005-0280-x 15711853 10.1007/s00702-005-0280-x

[37] Wang J, You H, Liu JF, Ni DF, Zhang ZX et al.. (2011) Association of olfactory bulb volume and olfactory sulcus depth with olfactory function in patients with Parkinson disease. Am J Neuroradiol 32: 677–81. doi 10.3174/ajnr.A2350. 10.3174/ajnr.A2350 PMC7965889 21330398

[38] Brodoehl S, Klingner C, Volk GF, Bitter T, Witte OW, et al (2012) Decreased olfactory bulb volume in idiopathic Parkinson’s disease detected by 3.0-Tesla magnetic resonance imaging. Mov Disord 27: 1019–1025 doi:10.1002/mds.25087 22730050 10.1002/mds.25087

[39] Acebes A, Martín-Peña A, Chevalier V, Ferrús A (2011) Synapse loss in olfactory local interneurons modifies perception. J Neurosci 31: 2734–2745 doi:–10.1523/JNEUROSCI.5046–10.2011 21414896 10.1523/JNEUROSCI.5046-10.2011 PMC6623785

[40] Greene AW, Grenier K, Aguileta MA, Muise S, Farazifard R, et al (2012) Mitochondrial processing peptidase regulates PINK1 processing, import and Parkin recruitment. EMBO Rep 13: 378–385 doi:10.1038/embor.2012.14 22354088 10.1038/embor.2012.14 PMC3321149

